# Vitamin D and Public Health

**DOI:** 10.3390/ijerph16050848

**Published:** 2019-03-08

**Authors:** David Scott, Peter R. Ebeling

**Affiliations:** 1 Department of Medicine, School of Clinical Sciences at Monash Health, Monash University, Clayton, Victoria 3168, Australia; peter.ebeling@monash.edu; 2 Department of Medicine and Australian Institute of Musculoskeletal Science, Melbourne Medical School—Western Campus, The University of Melbourne, St. Albans, Victoria 3121, Australia

Since the early 2000’s, interest in vitamin D has grown significantly among the research, clinical and lay communities. This has resulted in exponential increases in research outputs, clinical testing of vitamin D status, and availability of vitamin D supplements and fortified foods [1]. Despite this, vitamin D deficiency remains an important global public health issue, prevalent across many countries, cultures, and age groups [2]. There does, however, remain significant controversy regarding vitamin D, with insufficient evidence currently available to support a need for routine screening, or to explain its role in maintaining non-skeletal health [3]. Thus, as further research is required to achieve consensus on the role of vitamin D in public health, we were motivated to contribute to the scientific discourse through this Special Issue of the *International Journal of Environmental Research and Public Health*.

In this Special Issue, a systematic review by Heath et al. provided up-to-date evidence on the relationship between vitamin D and mortality. The authors concluded that 25-hydroxyvitamin D (25(OH)D) concentrations (up to a certain threshold) are associated with all-cause mortality, with limited evidence on their effect on cardiovascular mortality, but potential moderate effects on deaths due to respiratory diseases and cancer [4]. In support of these findings, a randomised controlled trial in Taekwondo athletes reported that increases in 25(OH)D concentrations in response to vitamin D supplementation are associated with reductions in upper respiratory tract infections during winter [5]. On the other hand, a separate review indicated that the current evidence from human studies does not support a role of vitamin D in reducing incidence of cancer, despite its potential benefit for cancer mortality and pre-clinical studies demonstrating that vitamin D may prevent carcinogenesis, tumour invasiveness and metastases [6]. Similarly, patients with chronic kidney disease who have adequate vitamin D concentrations appear to have a better prognosis for several outcomes including mortality, but the mechanisms for these effects are unclear and currently there is a lack of randomised controlled trials to support guidelines for prescribing vitamin D in this population [7]. Clearly, further research is required to determine how low vitamin D concentrations contribute to increased prevalence of some non-skeletal diseases, to rule out reverse causality as an explanation, and to elucidate the mechanisms by which they influence prognosis. 

Amongst non-skeletal effects of vitamin D, improvements in physical function have been amongst the most widely investigated, particularly in older adult populations. Our observational study presented evidence that low vitamin D status is common in overweight and obese older adults and also associated with poor quadriceps strength and lower-limb muscle power in women [8]. Observations such as this have led to numerous studies into the role of vitamin D supplementation for improving physical function and cardiometabolic health in older adults. A trial presented here provided preliminary evidence that vitamin D supplementation during an exercise program can reduce homocysteine (an amino acid linked with an increased risk of cardiovascular disease) concentrations in older women, although there was a lack of effect of vitamin D supplementation alone, which is consistent with evidence that exercise is more beneficial than vitamin D supplementation for cardiovascular health [9].

There is also substantial interest in vitamin D and women and children’s health as evidenced by several articles addressing this topic in this Special Issue. While it is currently unclear whether vitamin D supplementation improves fertility or pregnancy outcomes, vitamin D deficiency is common in pregnant and lactating women and supplementation may improve vitamin D and calcium status in the mother, foetus and infant [10]. From the Australian perspective, the review by Di Marco et al. highlighted that while cases of nutritional rickets are rare, children of newly-arrived immigrants in particular may be at increased risk, and so vitamin D deficiency remains a concern, even in a country with relatively high levels of sun exposure [11]. This was supported by an original research study of pre-school children in southern Croatia which observed that over half the study population had low 25(OH)D concentrations (with girls at a greater risk compared with boys), despite the high sun exposure levels in this region [12].

A significant challenge to improving vitamin D status on the population level is addressing poor knowledge and/or indifferent attitudes. Amongst UK adults, only half expressed concerns regarding their vitamin D concentrations, but over 80% wanted to learn more about vitamin D. Importantly, greater concern and knowledge predicted increased likelihood of vitamin D supplement use, while participants residing in England were three times less likely to be taking vitamin D compared with those in Scotland [13]. Similar findings were reported in a survey of Polish mothers; maternal education levels were significant predictors of knowledge about vitamin D and likelihood of using supplements [14]. Education and public health messaging are, therefore, key to ensuring individuals take steps to monitor and address their vitamin D status and that of their children. Finally, one of the most potentially effective strategies to improve the vitamin D status of populations is food fortification. Kiely and Cashman provided a compelling summary of the ODIN project, including almost 56,000 EU residents, reporting evidence that fortification strategies could safely increase intakes of vitamin D and prevent low 25(OH)D concentrations in a range of population sub-groups, including those at greatest risk [15].

In summary, this Special Issue demonstrated that low vitamin D concentrations are prevalent across populations and associated with increased mortality, reduced physical function, and poor prognosis for a variety of chronic disease conditions. Further research is required, however, to determine whether low vitamin D itself is a causative factor in these outcomes. Regardless, given its known benefits for skeletal health, particularly in children, future studies should explore methods for delivering education and food-fortification to a range of culturally and ethnically diverse populations in order to reduce the high prevalence of vitamin D deficiency worldwide. 
